# Effects of NH_4_F and distilled water on structure of pores in TiO_2_ nanotube arrays

**DOI:** 10.1038/s41598-018-30668-3

**Published:** 2018-08-21

**Authors:** Jaegyu Kim, Bongsoo Kim, Chungik Oh, Jeongjae Ryu, Hongjun Kim, Eugene Park, Kwangsoo No, Seungbum Hong

**Affiliations:** 1 Department of Materials Science and Engineering, KAIST, Daejeon, 34141 Republic of Korea; 2 Materials and Energy Science and Engineering, Nelson Mandela African Institute of Science and Technology, Arusha, 447 Tanzania

## Abstract

In this study, we report the influences of distilled water and ammonium fluoride (NH_4_F) on morphology of pores in honeycomb-like titanium dioxide (TiO_2_) nanotube arrays. We observed the structure and arrangement of pores in the TiO_2_ nanotube arrays based on scanning electron microscopy images and analyzed the spatial distribution of the pores using fast Fourier transform and Voronoi diagram. We studied the individual pore properties including pore diameter, wall thickness, and interpore distance and found that locally connected ordering defects decreased with increasing distilled water concentration. Furthermore, we found that the optimum conditions of well-ordered hexagonal pore arrangement were 2 and 10 vol% distilled water with 0.2 and 0.4 wt% NH_4_F, respectively. Throughout this study, we provide a better understanding about the roles of distilled water and NH_4_F in forming well-ordered nanoscale pore structure with less ordering defects in the honeycomb-like TiO_2_ nanotube arrays.

## Introduction

TiO_2_ nanotube arrays have gained a lot of interest because their high surface area and effective separation^1–5^ for both photo-electrons and photo-holes are attractive for a variety of applications like photocatalysts^6,7^, solar cells^8,9^, gas sensors, supercapacitors^10,11^, and filtering systems^12^. The morphology, length and geometry of the TiO_2_ nanotube arrays strongly affect their performance, such as the efficiency of photocatalysis. For example, specific surface area, controlled by porosity, affects the photocurrents of the TiO_2_ nanotube arrays^13^. It is worth noting that electrolyte, concentrations of F^−^ ion and distilled water, applied voltage, anodizing time, pH and temperature can control the dimension and the morphology of the nanotubes^14–24^. This is important because if we can control the morphology, we can custom-tailor the TiO_2_ nanotube arrays for the aforementioned applications.

As such, many groups have studied the effects of the above-mentioned factors on the shape, size, and properties of the TiO_2_ nanotube arrays during the anodization^23–26^. For example, González *et al*.^27^ and Kojima *et al*.^17^ analyzed the effects of the concentrations of water and F^−^ ion on the diameter, length and anodization rate of the TiO_2_ nanotube arrays. Other groups characterized the effects of anodizing time^20^ and applied voltage^28^ on the structural properties of the nanotubes. However, there are few reports on the effects of additives in the organic electrolyte on the hexagonal ordering of the pores during the anodization. The hexagonal ordering is the closest stacking structure of pores in two-dimension, which means that the pores are arranged uniformly in the closest form. If the TiO_2_ nanotube arrays have a specific functional layer on the surface of the nanotubes, the close packed hexagonally arranged pores, which form the most efficient channel structure, can be used in photocatalysis and self-cleaning systems^12^. To elucidate this effect, we analyzed the influences of the concentrations of NH_4_F and distilled water on the pore distribution of the TiO_2_ nanotube arrays.

Here, we visualize the pore distribution using Voronoi diagram and fast Fourier transform (FFT). In addition, we analyze the morphology of individual pores. To understand the hexagonal ordering of the pores, we propose the mechanism on the formation of the ordering defects. We also present the optimal concentrations of the ethylene glycol electrolyte for uniform distribution of the pores.

## Methods

### Fabrication of TiO_2_ nanotube arrays

We firstly cut a commercially available Ti foil (99.5% purity, thickness: 0.2 mm, Nilaco, Tokyo, Japan) into small Ti foils with an area of 1 × 2 cm^2^ and cleansed them in acetone and ethanol with ultrasonication for 1 hour. Then we rinsed them in distilled water for 1 hour and dried them in air for 1 hour. For an actual area of 1 cm^2^, we put nail polish on backs and sides of the Ti foils. We fabricated TiO_2_ nanotube arrays on the small Ti foils in ethylene glycol electrolyte containing 0.2 and 0.4 wt% NH_4_F and 2, 4, 6, 8 and 10 vol% distilled water at 60 V using a DC power supply (OPE-1505DI, ODA Technologies, Incheon, Republic of Korea) for 1 hour by two-step anodization in ambient condition. We used a round Pt electrode, made by whirling a Pt wire, as a counter electrode as shown in the Supplementary Fig. S1(b).

The firstly fabricated TiO_2_ nanotube arrays were removed in distilled water after ultrasonication for 1 hour leaving concave patterns on the Ti foils. We secondly fabricated the TiO_2_ nanotube arrays on patterned Ti foils in the same electrolytes using the same conditions described above. After the second anodization, the TiO_2_ nanotube arrays were rinsed in distilled water for 1 hour to remove the remaining electrolyte and were dried in air for 1 hour. To form anatase phase, the samples were annealed at 450 °C in a muffle furnace (Isotemp Programmable Muffle Furnace 650, Fisher Scientific, Hampton, USA) for 1 hour with heating and cooling rates of 2 °C/min.

### Characterization

We characterized the surface morphology of as-fabricated TiO_2_ nanotube arrays by field emission scanning electron microscopy (FE-SEM, Hitachi S-4800, Hitachi Ltd., Tokyo, Japan). To make the Voronoi diagrams, we used Image J 1.49 v (Freeware) and Photoshop 13.0 v (Adobe). We adopted WSxM 5.0 Develop 8.2 software (Freeware) for fast Fourier transform (FFT) images and three diagonal lines of the FFT images. Pore properties, such as pore diameter, interpore distance, wall thickness, porosity, pore density, defect ratio, coordination number, regularity ratio and circularity, were analyzed using Image J 1.49 v. In addition, we analyzed X-ray diffraction (XRD) patterns using Jade 5.0 (see Supplementary Fig. S2).

## Results and Discussion

We investigated the effects of the concentrations of NH_4_F and distilled water on microstructure of the pores, configuration of defects, hexagonal pore arrangement, and pore size distribution, wall thickness as well as interpore distance.

**Figure 1:**
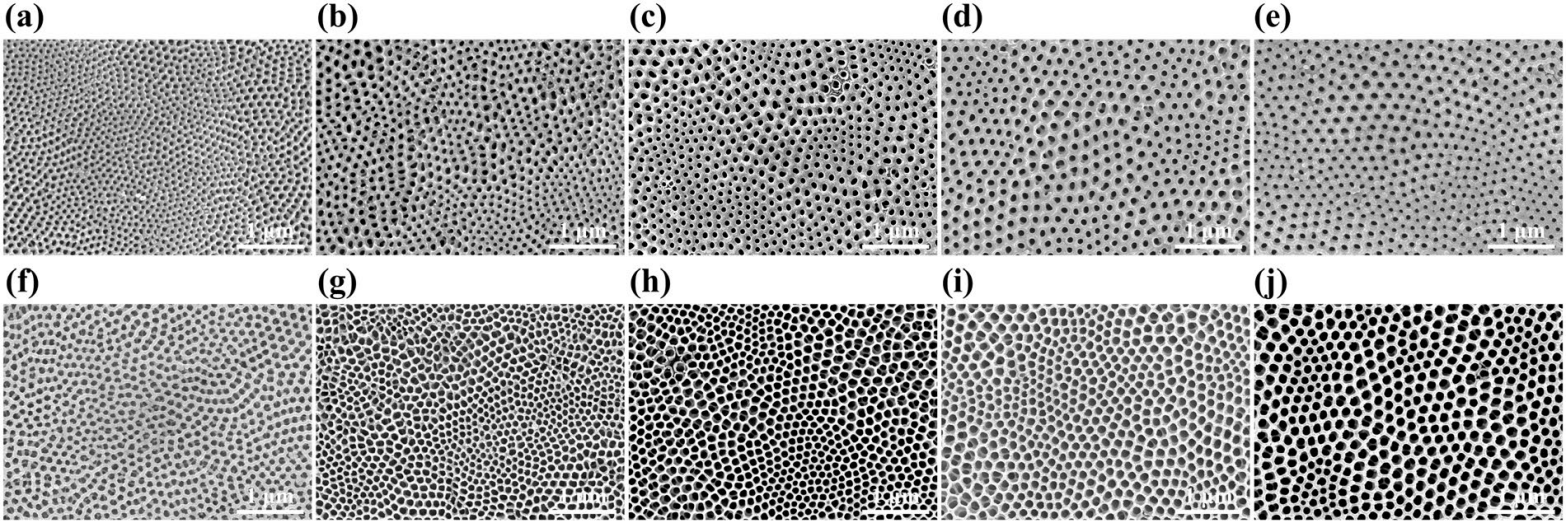
Top-view SEM images of TiO_2_ nanotube arrays anodized at 60 V for 1 hour in ethylene glycol containing **(a–e)** 2, 4, 6, 8, 10 vol% distilled water with 0.2 wt% NH_4_F and **(f–j)** 2, 4, 6, 8, 10 vol% distilled water with 0.4 wt% NH_4_F.

Figure 1(a–j) shows top-view SEM images of the TiO_2_ nanotube arrays fabricated in the ethylene glycol electrolyte with different concentrations ranging from 2 to 10 vol% of distilled water and from 0.2 to 0.4 wt% of NH_4_F. Initially, we chose the concentration ranges of NH_4_F and distilled water based on the previously reported conditions of well-ordered TiO_2_ nanotube arrays^17^. As such, we covered the range of NH_4_F from 0.2 wt% (0.06 M) to 0.8 wt% (0.24 M) (see Supplementary Table S1). However, for the conditions of 0.5, 0.6 and 0.8 wt% of NH_4_F and 12 vol% of distilled water, all the nanotubes collapsed or contained large-scale cracks on the surface.

From Fig. 1, we can see that the pores of all samples are hexagonally arranged to form honeycomb-like structure (see Supplementary Fig. S3 for higher magnification). This is because the TiO_2_ nanotube arrays are connected to each other in a close packed manner by the two-step anodization^29^. More detailed growth mechanism of the two-step anodization will be discussed in the next section.

We can observe typical grain boundaries where each grain consists of pores with the same arrangement if we imagine that the pores represent crystal lattices. Only in the samples with 2 vol% of distilled water, some of the edges are not connected, and the disconnected areas have the coalescence of pores in a complex manner. This may be due to the incomplete detachment of TiO_2_ nanotube arrays initially fabricated from the Ti foil during the ultrasonic treatment.

We measured the porosity and the pore density of the hexagonally arranged pores from the SEM images. Samples which were anodized in 0.4 wt% NH_4_F have much larger areas of pores than those which were fabricated in 0.2 wt% NH_4_F (see Supplementary Information, Fig. S4(a)). This difference can be attributed to the higher chemical dissolution of TiO_2_ film by higher concentration of F^−^ ions at 0.4 wt% NH_4_F^13^. The trend of porosity with respect to the water content is different for 0.2 wt% and 0.4 wt% NH_4_F. For 0.2 wt% NH_4_F, the porosity decreases as the water content increases, however, for 0.4 wt% NH_4_F, it initially increases until 6 vol% distilled water from which it decreases as the water content increases. The trends of pore density as a function of water content are more similar for 0.2 wt% and 0.4 wt% NH_4_F where the pore density decreases as the distilled water concentration increases (see Supplementary Information, Fig. S4(b)).

**Figure 2:**
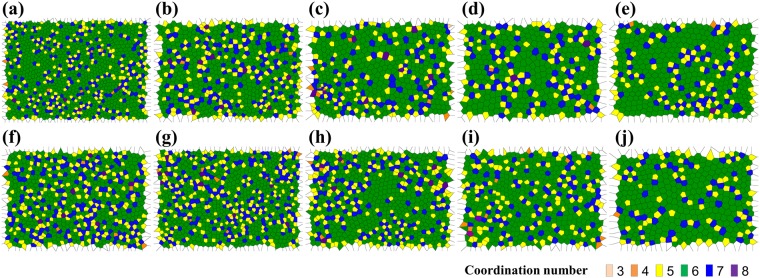
Voronoi diagrams of TiO_2_ nanotube arrays anodized at 60 V for 1 hour in ethylene glycol containing **(a–e)** 2, 4, 6, 8, 10 vol% distilled water with 0.2 wt% NH_4_F and **(f–j)** 2, 4, 6, 8, 10 vol% distilled water with 0.4 wt% NH_4_F. Coordination numbers of cells are expressed in colors: rachel: 3, orange: 4, yellow: 5, green: 6, blue: 7, violet: 8.

The Voronoi diagram of pores are shown in Fig. 2(a–j). To make the Voronoi cells, we constructed the polygons by connecting the central points of neighboring pores in the SEM images (Fig. 1(a–j)). To clarify the coordination number of each pore, we colored each cell with a color of rachel, orange, yellow, green, blue, and violet corresponding to the coordination numbers from 3 to 8.

The Voronoi diagram has dual relationship with Delaunay tessellation, which means that we can derive the former with the latter, and vice versa^30,31^. Three center points of three pores form a triangle, and the arrays of triangles form the Delaunay tessellation. S. Mátéfi-Tempfli *et al*.^30^ reported the Delaunay tessellation carried out on nanoporous anodic aluminum oxide (AAO). Both methods enable us to visualize the configuration of defects.

While most pores had the coordination of 6 forming a hexagonal matrix, most defects had the coordination number of either 5 (yellow) or 7 (blue) forming dendritic features by alternately connecting to each other as shown in Fig. 2.

Our hypothesis for these features is as follows: we suppose that there is a certain area where two neighboring hexagons can be located^32^. If 5 pores form a pentagon in a certain area of interest, they have smaller area than that of a hexagon leading to an extra space. In the remaining area with the additional space, more pores can form a polygon such as a heptagon. This heptagon induces another pentagon, which leads to another heptagon. For this reason, the sequence of 5, 7, 5, 7, … can be generated. This trend was previously identified in anodic aluminum oxides^33^.

In order to understand why defects with coordination number of either 5 or 7 nucleate, we analyzed the chemical reactions involved in the growth of TiO_2_ nanotube arrays. The growth reactions of TiO_2_ nanotube arrays include field-assisted oxidation, chemical dissolution, field-assisted dissolution, and field-assisted ejection of Ti^20,34^. For the oxidation, Ti releases electrons at the anode as shown in Eq. (1).

1 $${\rm{Ti}}\to {{\rm{Ti}}}^{4+}+4{{\rm{e}}}^{-}$$

The released Ti^4+^ ions combine with O^2−^ ions and OH^−^ ions to create oxide and hydrated layer (Eqs. (2 and 3)). O^2−^ and OH^−^ ions are dissociated from water by high electric field. Ti(OH)_4_ also becomes TiO_2_ releasing water by a condensation reaction (Eq. (4)).

2 $${{\rm{Ti}}}^{4+}+2{{\rm{O}}}^{2-}\to {{\rm{TiO}}}_{2}$$

3 $${{\rm{Ti}}}^{4+}+4{{\rm{OH}}}^{-}\to {\rm{Ti}}{({\rm{OH}})}_{4}$$

4 $${\rm{Ti}}{({\rm{OH}})}_{4}\to {{\rm{TiO}}}_{2}+2{{\rm{H}}}_{2}{\rm{O}}$$

At the cathode, hydrogen evolution occurs with hydrogen ions and electrons (Eq. (5)).

5 $$4{{\rm{H}}}^{+}+4{{\rm{e}}}^{-}\to 2{{\rm{H}}}_{2}({\rm{g}})$$

As an overall reaction (Eqs. (1–4)), Ti reacts with water to create oxide and hydrogen gas (Eq. (6)).

6 $${\rm{Ti}}+2{{\rm{H}}}_{2}{\rm{O}}\to {{\rm{TiO}}}_{2}+2{{\rm{H}}}_{2}({\rm{g}})$$

In chemical dissolution, F^−^ ions in the electrolyte dissolve the oxide and the hydrated layer, which suppress the growth of the oxide (Eqs. (7 and 8)).

7 $${{\rm{TiO}}}_{2}+6{{\rm{F}}}^{-}+4{{\rm{H}}}^{+}\to {{{\rm{TiF}}}_{6}}^{2-}+{{\rm{H}}}_{2}{\rm{O}}$$

8 $${\rm{Ti}}{({\rm{OH}})}_{4}+6{{\rm{F}}}^{-}\to {{{\rm{TiF}}}_{6}}^{2-}+4{{\rm{OH}}}^{-}$$

F^−^ ions also react with Ti^4+^ ions within the oxide by electric field (Eq. (9)).

9 $${{\rm{Ti}}}^{4+}+6{{\rm{F}}}^{-}\to {{{\rm{TiF}}}_{6}}^{2-}$$

It should be noted that Ti^4+^ can be created from Ti-O bonds by electric field (field-assisted dissolution). As a side reaction, the evolution of oxygen occurs at the anode (Eq. (10)). This oxygen evolution creates oxide rings and ribs on the wall of the nanotubes and affects the growth efficiency^35^.

10 $$2{{\rm{H}}}_{2}{\rm{O}}\to {{\rm{O}}}_{2}+4{{\rm{e}}}^{-}+4{{\rm{H}}}^{+}$$

Oxidation of Ti foil surface via chemical reaction between Ti and H_2_O leads to compressive state due to the volume expansion of TiO_2_ which is confined by the Ti substrate. TiO_2_ surface layer reacts with incoming NH_4_F to create pits that are the source of the pores. Pores will maintain a certain distance between them due to the repulsive interaction created by the compressive stress field inside TiO_2_ film (see Supplementary Information, Fig. S5(a)). This radially symmetric repulsive interaction favors high symmetry and close packed structure, which results in hexagonal arrangement. The hexagonal ordering of pores is governed by the balance of the stress^33^. The magnitude of the repulsive stress would locally be different. The larger repulsive stress than the average stress would make the distance between pores longer and the smaller repulsive stress would affect the distance smaller. A pentagon could appear from the former condition and a heptagon could appear from the latter one. In the Voronoi diagrams, pentagons and heptagons are alternately connected, which means that larger and smaller stresses are adjoining^33^.

The adhesion between TiO_2_ nanotube arrays and Ti substrate is weak because of the formation of fluoride-rich layer at the interface of Ti and TiO_2_ nanotubes^36^. This comes from the fact that migration rate of F^−^ ions is twice that of O^2−^ ions through the TiO_2_ layer. In the first anodization, the TiO_2_ nanotube arrays grow through three stages. First, compact TiO_2_ layer is formed on the Ti substrate. Second, pores are formed on the TiO_2_ layer. As the pores grow perpendicularly to the TiO_2_ film, voids grow between the pores and they also grow perpendicularly to the TiO_2_ film. After enough anodization time, pores and voids become very long and they form nanotubes. In the last stage, TiO_2_ nanotubes grow longer with time. The ultrasonic treatment detaches the TiO_2_ nanotubes from the Ti foil leaving concave textured patterns on the Ti substrate^29^. The patterns act as nucleation sites for pores, and more hexagonally ordered pores and vertically aligned nanotubes are formed^25^. During the second anodization, TiO_2_ film has the concave surface which enhances the electric field from the anode to the cathode (see Supplementary Information, Fig. S5(b)). This increased electric field supports the selective etching of the TiO_2_ film with higher TiF_6_^2−^ ions at the concave valleys. As a result, the pores more easily form on the hexagonally ordered concave valleys. However, the voids grow at a certain distance from the top surface of the TiO_2_ film. Due to this method, we can fabricate edge-connected honeycomb-like TiO_2_ nanotube arrays similar to prior reports^29^.

Defect ratio is defined as the ratio of the number of pores with a coordination number other than 6 to the total number of pores. Defect ratio can be explained by correlation with volume expansion and pore diameter. Jessensky *et al*.^37^ reported that for anodic aluminum oxide (AAO) case, moderate expansion of the aluminum during oxidation is most suitable for the hexagonal ordering of pores^38^.

We observed less defects in higher distilled water concentration for both 0.2 wt% and 0.4 wt% NH_4_F cases (see Supplementary Information, Fig. S6 and S7). As with the case of AAO, this may indicate that higher distilled water concentration leads to less volume expansion of the TiO_2_ film resulting in lower defect ratio. Albu *et al*.^39^ attributed the reason of smaller volume expansion to the higher water contents because of higher dissolution of Ti and TiO_2_ and lower growth efficiency. Higher dissolution with increasing water is caused by enhanced diffusion of H^+^ and F^−^ ions due to lower viscosity^40^, which therefore leads to larger pore diameter. On the other hand, NH_4_F contents had little influence on the defect ratio. This tendency is in accordance with prior findings reporting that the fluoride content has little effect on the volume expansion^41,42^.

The pore diameter may also affect the hexagonal ordering. The energy for a small pore to deviate from the ideal position is smaller than that of a large pore. This may be because a large pore not only has a longer (interpore) distance to deviate from the ideal position, but also experiences higher repulsive interaction from the neighboring pores.

**Figure 3:**
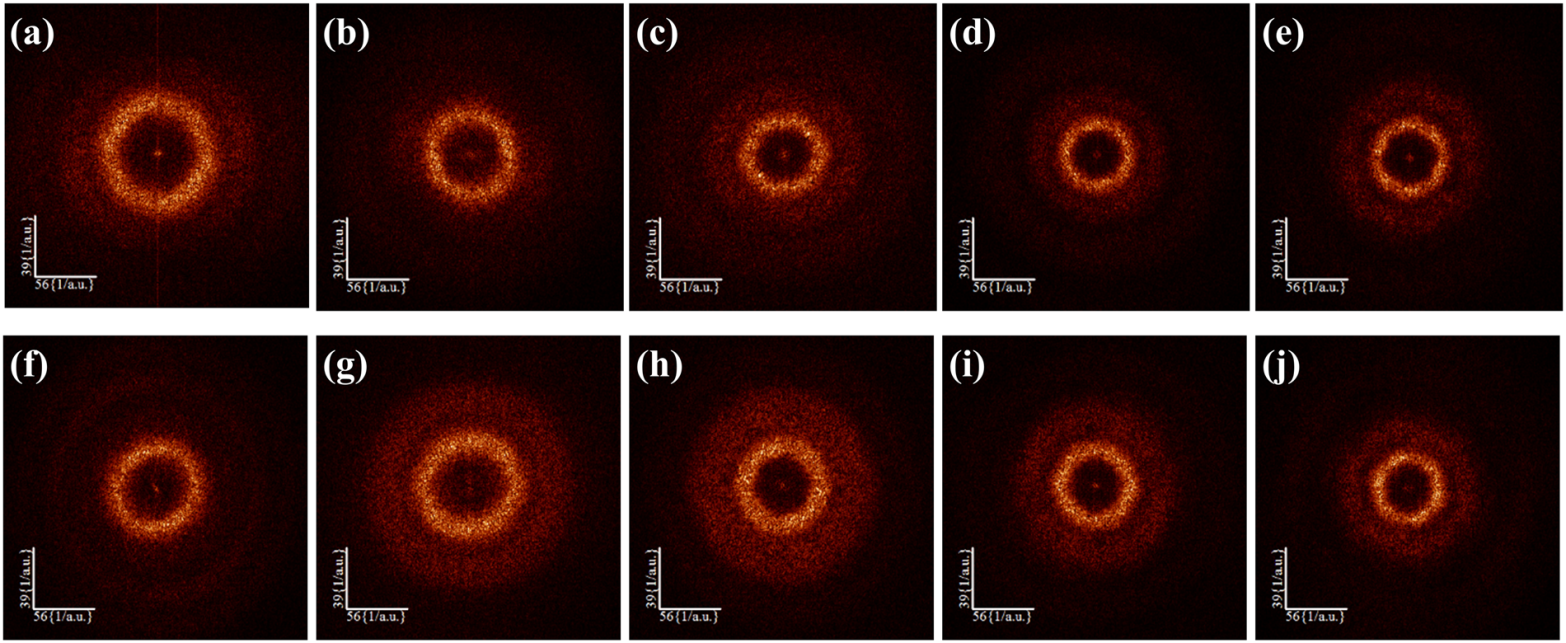
Fast Fourier transform images of TiO_2_ nanotube arrays anodized at 60 V for 1 hour in ethylene glycol containing **(a–e)** 2, 4, 6, 8, 10 vol% distilled water with 0.2 wt% NH_4_F and **(f–j)** 2, 4, 6, 8, 10 vol% distilled water with 0.4 wt% NH_4_F.

Figure 3(a–j) shows the FFT images obtained from the SEM images (Fig. 1(a–j)). The FFT images had either hexagonal rings or hexagon ring shaped contrasts^43,44^. The more hexagonality we see in the FFT image, the less the ordering defects will appear in the Voronoi diagram.

The FFT images demonstrate the degree of hexagonal ordering. The more hexagonally the pores are arranged, the more hexagonally the FFT ring is formed. The FFT image of the ideal hexagonal pore arrangement has a six-fold symmetry. If a hexagonal pore arrangement has short-distance periodicity and slightly disturbed long-range order, its FFT image has a thin hexagonal ring or even a disc-shaped form^43,44^.

The rings of all samples were not distorted unlike prior reports, even though the SEM images had a larger scale than those of Sulka *et al*.^43^. It may be attributed to the fact that the TiO_2_ nanotube arrays were fabricated by the two-step anodization for sufficient growth time. After the first anodization for 1 hour, the concave patterns were distributed more uniformly and hexagonally on the Ti foil.

We analyzed the hexagonal ordering of the samples from three diagonal lines of hexagons in the FFT images (see Supplementary Information, Figs S8, S9 and S10). The difference between the lengths of the three diagonal lines tended to decrease as the distilled water concentration increased.

The average FFT radius profiles, which show the distribution of the interpore distance, were derived from the FFT images (see Supplementary Information, Fig. S11(a–j)). The intensity of the maximum peak in the Supplementary Fig. S11 increases while its width decreases as the hexagonal arrangement becomes more uniform^45^. When compared with the prior reports^43,45^, all the samples showed higher intensities, indicative of more uniform arrangement regardless of the NH_4_F and distilled water contents. Within our samples, we found that the uniformity of arrangement improved as the water content increased based on the intensity and width of the maximum peak data.

In order to quantitatively study the uniformity of hexagonal arrangement, we calculated the regularity ratio (RR), which represents the regularity of the pore arrangement^43^ (Supplementary Information, Fig. S12(a and b)) from Eq. (11) where *I*_*max*_ is the maximum intensity of the peak, *W*_1/2_ is the width of the peak, and *D*_*ave*_ is the average interpore distance.

11 $${\rm{RR}}=\frac{{I}_{max}}{{W}_{1/2}\cdot {D}_{ave}}$$

The highest regularity ratio was observed in the samples with 10 vol% distilled water and 0.2 wt% NH_4_F concentrations and with 2 vol% distilled water and 0.4 wt% NH_4_F concentrations.

Based on our analysis of the shape of the pattern, the intensity and width of the peak, and regularity ratio, we found that the samples with 10 vol% of distilled water and both 0.2 wt% and 0.4 wt% of NH_4_F showed a well-ordered hexagonal contrast because they had relatively low defect ratio, low deviation of the three diagonal lines, thin hexagonal rings, and high regularity ratio.

**Figure 4:**
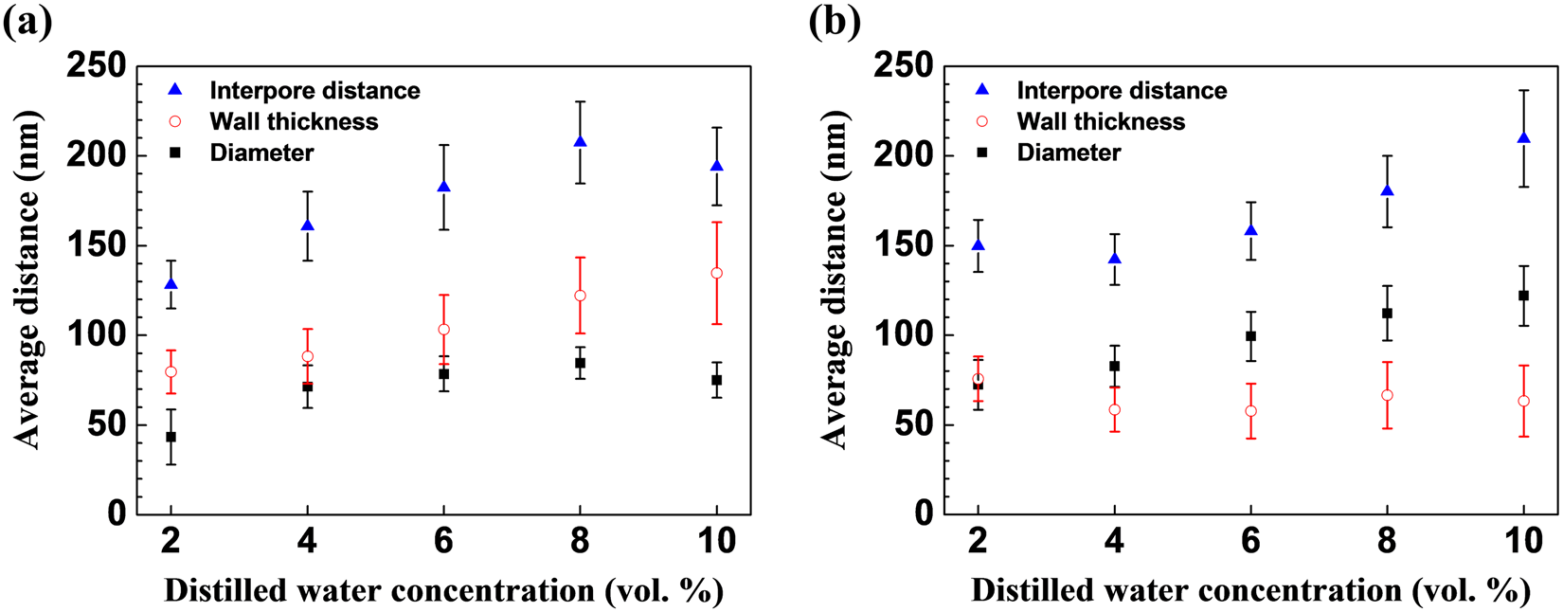
Average interpore distance, wall thickness, and diameter of TiO_2_ nanotube arrays anodized at 60 V for 1 hour in ethylene glycol containing **(a)** 0.2 wt% NH_4_F and **(b)** 0.4 wt% NH_4_F as a function of vol% of distilled water.

We investigated the pore size distribution, the average wall thickness as well as the average interpore distance as shown in Fig. 4. When NH_4_F was 0.2 wt% and 0.4 wt%, the average pore diameter increased from 43 to 75 nm and from 72 to 122 nm as distilled water increased, respectively. This tendency is in accordance with the tendency of the prior report^40^. This can be attributed to the fact that the increasing distilled water contents reduced the viscosity of the electrolyte resulting in enhancement of its diffusion coefficient. During anodization, F^−^ ions flow faster into the interface between the oxide and the Ti foil in the electrolyte with higher diffusion coefficient. As a result, more concentrated F^−^ ions participate in the chemical dissolution leading to bigger pores^40^.

The wall thickness is larger than the diameter when NH_4_F is 0.2 wt%, but the wall thickness is smaller than the diameter when NH_4_F is 0.4 wt% regardless of the distilled water content. The reason we see this reverse trend is because F^−^ ion in NH_4_F etches the wall leading to thinner wall thickness and larger pore diameter.

When NH_4_F was 0.2 wt% and 0.4 wt%, the average interpore distance increased from 128 to 194 nm and from 149 to 209 nm as distilled water concentration increased from 2 to 10 vol%, respectively. The average interpore distance is inversely proportional to the pore density according to Eq. S2 in the Supplementary Information. While the average interpore distance tended to increase, the pore density tended to decrease as distilled water concentration increased from 2 to 10 vol% in both 0.2 and 0.4 wt% NH_4_F cases.

The standard deviations for interpore distance and wall thickness are relatively high for the samples with high water concentrations. This is due to the larger variation of local chemical reaction rate, i.e. chemical dissolution, in higher concentration of water. According to the Stokes-Einstein relation, the diffusion coefficients of H^+^ and F^−^ ions increase with increasing water concentration. This amplifies local ionic concentration fluctuations and pH bursts during anodization, resulting in irregularity of dissolution rate and side wall profiles^46^. Therefore, the standard deviations for interpore distance and wall thickness are high for samples with higher concentration of water. Although the polygons of the samples with high water concentrations have more distorted shapes due to the high standard deviation, the majority of them are still hexagons as shown in Fig. 2, which leads to the close packed structure.

Based on the trends of the pore diameter and the shape of the pore represented by the regularity in the Supplementary Fig. S12, we can understand the relationship between the ordering defect ratio, the pore diameter and the regularity. When the regularity ratio is higher and the diameter is larger, the defect ratio becomes lower. We induced the trends of defect ratio from regularity ratio and diameter by line fitting as shown in the Supplementary Fig. S13. After combining the induced two trends of defect ratio, we found that the trends are similar with the results of line fitting from defect ratios of the Supplementary Fig. S7.

Finally, we calculated the circularity of individual pores from the SEM images as shown in the Supplementary Fig. S14. The circularity close to 0 indicates that the pore is an elongated polygon, and the circularity of 1.0 means that the pore is ideally circular^47^. All samples had the average circularity higher than 0.6^48^, and the circularity tended to increase as distilled water concentration increased. The increase in the circularity originates from the enhanced field-assisted isotropic chemical dissolution at the interface between the oxide and the electrolyte by the increase in distilled water concentration^44^. This result is consistent with the tendency of more uniform hexagonal arrangement as distilled water concentration increased.

## Conclusions

In conclusion, we analyzed the effects of distilled water and NH_4_F concentration in the ethylene glycol electrolyte on the pore structure of the TiO_2_ nanotube arrays. The defect ratio decreased with increasing distilled water concentration, and the defects were locally connected together. The well-ordered hexagonal pore arrangement was observed in 2 vol% and 10 vol% distilled water with 0.2 wt% and 0.4 wt% NH_4_F, respectively, but the regularity was improved by increasing the distilled water concentration. The pore diameter changed from 43 to 122 nm with more than 0.65 of the circularity when distilled water concentration increased. Throughout this study, we provide a better understanding about the role of distilled water and NH_4_F concentration in forming a well-ordered nanoscale pore structure with less defects.

## Electronic supplementary material

Supplementary Information

## Data Availability

The datasets generated and analyzed during the current study are available from the corresponding author on reasonable request.
