# Supplementary material for: Effects of NH4F and distilled water on structure of pores in TiO2 nanotube arrays
Source: Sci Rep. 2018 Aug 21;8:12487. doi: 10.1038/s41598-018-30668-3 (PMC6104029; doi:10.1038/s41598-018-30668-3)
Supplement: Supplementary file 1 — Supplementary Information [file 41598_2018_30668_MOESM1_ESM.docx]

**Supplementary Information**

**Effects of NH_4_F and distilled water on structure of pores in TiO_2_ nanotube arrays**

Jaegyu Kim^1^, Bongsoo Kim^1^, Chungik Oh^1^, Jeongjae Ryu^1^, Hongjun Kim^1^, Eugene Park^2^, Kwangsoo No^1^ and Seungbum Hong^1,*^

^*^Corresponding author: [seungbum@kaist.ac.kr](mailto:seungbum@kaist.ac.kr)

^1^Department of Materials Science and Engineering, KAIST, Daejeon 34141, Republic of Korea

^2^Materials and Energy Science and Engineering, Nelson Mandela African Institute of Science and Technology, Arusha 447, Tanzania

Fig. S1(a – d) shows the digital images of the results of the first anodization in the ethylene glycol electrolyte containing 0.2 and 0.4 wt. % NH_4_F without distilled water. Fig. S1(a) shows the digital image of the Pt counter electrode. Fig. S1(b) shows the digital image of the Ti foil anodized in the electrolyte containing 0.2 wt. % NH_4_F for 1 hour. There was almost no change on the Ti foil after the first anodization due to the absence of distilled water.^1^ After 80 seconds of the first anodization in the electrolyte containing 0.4 wt. % NH_4_F, gas flowed from the electrolyte, of which color changed from transparent color into pale ruby one as shown in Fig. S1(c). Furthermore, the Ti foil was much etched losing some parts of it as shown in Fig. S1(d). This result might be caused by much excessive etching because there was no water, which is indispensable for anodization^1^.


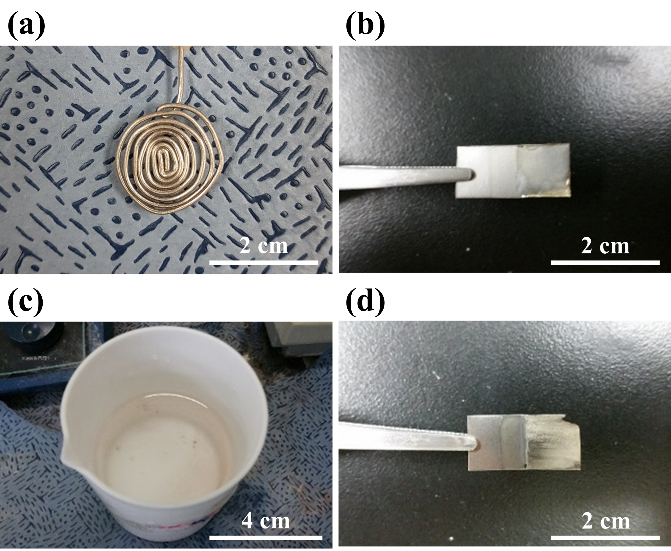


**Figure S1**. Digital images of the first anodizing time (a) for 80 seconds at 60 V in ethylene glycol containing 0.4 wt. % NH_4_F with 0 vol. % distilled water and (b) the electrolyte and (c) the Ti foil after the anodization. Digital image of the first anodized Ti foil at 60 V for 1 hour in ethylene glycol containing (d) 0.2 wt. % NH_4_F with 0 vol. % distilled water.

We collected X-ray diffraction (XRD) patterns with 2θ ranging from 20 to 80 degrees using a X-ray diffractometer (XRD model Rigaku Ultima Ⅳ, Tokyo, Japan) with Cu Kα radiation (λ=0.15406 nm). XRD peaks show that the TiO_2_ nanotube arrays have anatase phase as shown in Fig. S2. The TiO_2_ nanotube arrays contained peaks (101), (004), (200), (105), (211), (204), (116), (220) and (215) of anatase phase (PDF#21-1272, JCPDS)^2^.


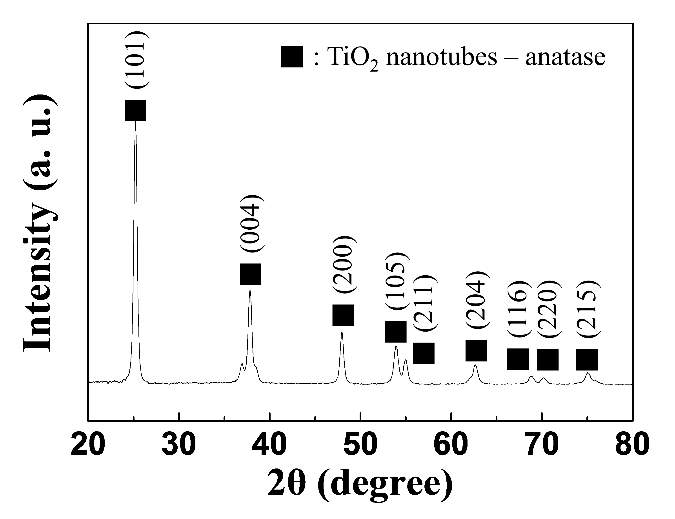


**Figure S2**. XRD patterns of TiO_2_ nanotube arrays.

Table S1 presents morphologies of the TiO_2_ nanotube arrays. Nanowires, nanoparticles, and detached honeycomb-like nanochannels cover the top surface of the TiO_2_ nanotube arrays fabricated in the ethylene glycol electrolyte with concentration of more than 0.4 wt. % NH_4_F. The excessive contents of NH_4_F broke down the structure of the TiO_2_ nanotube arrays due to the excessive dissolution^3,4^. Likewise, above 10 vol. % of distilled water, the structure was collapsed, so the maximum distilled water that we can put is 10 vol. %^5,6^. For better ordering of the pores in the TiO_2_ nanotube arrays, Ti foil needs to be chemically or mechanically etched for a clean surface^7^. Additional ultrasonic treatment and third anodization can make clean and better hexagonal ordering of the pores in the TiO_2_ nanotube arrays^8^. Our Ti foil samples might have quite rough surface because we did not treat Ti foil by the aforementioned methods.

Fig. S3(a – j) shows top-view SEM images of the TiO_2_ nanotube arrays fabricated in the ethylene glycol electrolyte with different concentrations ranging from 2 to 10 vol. % of distilled water and from 0.2 to 0.4 wt. % of NH_4_F in higher magnification of ×150,000 than the magnification of ×25,000 of Fig. 1(a – j). We found that the shape of pores in samples with 2 vol. % distilled water are much more distorted than those of the other samples.

Table S1. Morphologies of TiO_2_ nanotube arrays grown in different electrolyte compositions. X corresponds to conditions that we did not fabricate.

|  | | NH_4_F (wt. %) | | | | | |
| --- | --- | --- | --- | --- | --- | --- | --- |
|  |  | 0.2 | 0.3 | 0.4 | 0.5 | 0.6 | 0.8 |
| Distilled water (vol. %) | 2 | Well-ordered | Well-ordered | Well-ordered | Disorganized   | Bundles   | Bundles   |
|  | 4 | Well-ordered | Well-ordered | Well-ordered | Disorganized   | Disorganized   | Disorganized   |
|  | 6 | Well-ordered | X | Well-ordered | X | Bundles   | Bundles   |
|  | 8 | Well-ordered | X | Well-ordered | X | Cracks   | Bundles   |
|  | 10 | Well-ordered | X | Well-ordered | X | Bundles   | X |
|  | 12 | X | X | Collapsed   | X | X | X |


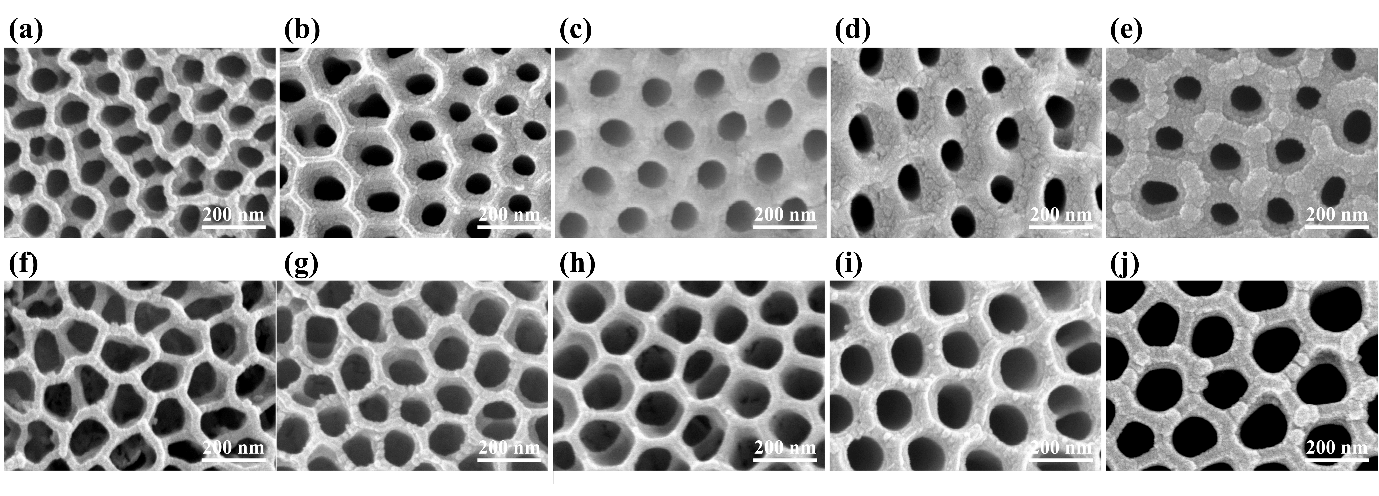


**Figure S3**. Top-view SEM images of TiO_2_ nanotube arrays anodized at 60 V for 1 hour in ethylene glycol containing (a – e) 2, 4, 6, 8, 10 vol. % distilled water with 0.2 wt. % NH_4_F and (f – j) 2, 4, 6, 8, 10 vol. % distilled water with 0.4 wt. % NH_4_F. The scale bar is 300 nm.

Fig. S4(a) presents porosity ($\alpha$) and (B) pore density (n) of the TiO_2_ nanotube arrays fabricated in the ethylene glycol electrolyte with 0.2 and 0.4 wt. % NH_4_F. The porosity is defined as the ratio of area of pores to the total area, and the pore density is defined as the number of pores on the surface area of 1 cm^2^ for an ideal hexagonal pore structure. We calculated the porosity and pore density by Equations (1) and (2) which are discussed in previous reports^9,10^.

$\alpha$ =${\frac{\pi}{2\sqrt{3}} \cdot\left( \frac{D_{p}}{D_{i}} \right)}^{2}$ (1)

n = $\frac{2\cdot{10}^{14}}{\surd3\cdot D_{i}^{2}}$ (2)

where $D_{p}$is the average diameter of the pores, and $D_{i}$is the average interpore distance. The samples with 0.4 wt. % NH_4_F have two times higher porosity than that of the samples with 0.2 wt. % NH_4_F, which means that much larger portions of the total area of samples with 0.4 wt.% NH_4_F are composed of the pores than those of the samples with 0.2 wt. % NH_4_F.

The pore density tends to decrease when distilled water concentration increases, which corresponds to that the average interpore distance tends to increase according to the increase of distilled water (see Fig. 4(a) and (b)).

**
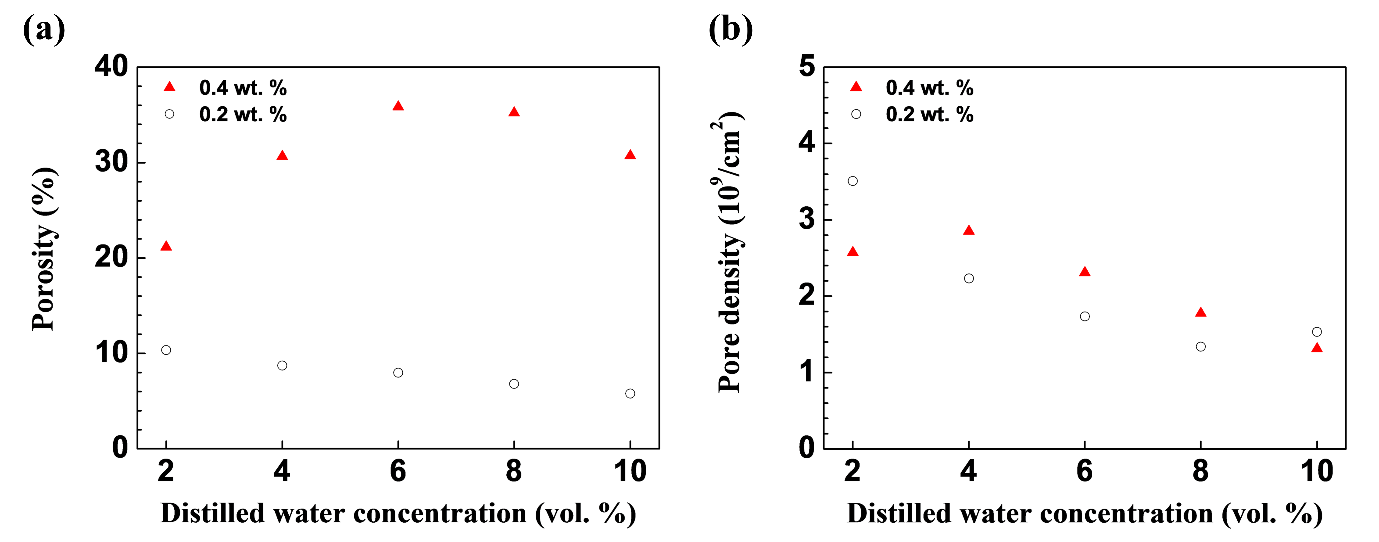
Figure S4**. Porosity and pore density of TiO_2_ nanotube arrays anodized at 60 V for 1 hour in ethylene glycol containing (a) 0.2 wt. % NH_4_F and (b) 0.4 wt. % NH_4_F as a function of vol. % of distilled water.

Fig. S5 shows the schematic of the first and second anodization processes. The first anodization starts from formation of TiO_2_ film on Ti foil and pits on the TiO_2_ film. Pores grow from the initial pits and voids form between pores. Along the pores and voids, nanotubes form and grow. After the nanotubes are detached from the Ti foil, it has a concave surface. The second anodization starts from the formation of TiO_2_ film on the concave surface of the Ti foil. Pits and pores again grow and voids also appear. Along the pores and voids, honeycomb-like TiO_2_ nanotube arrays finally form and grow.


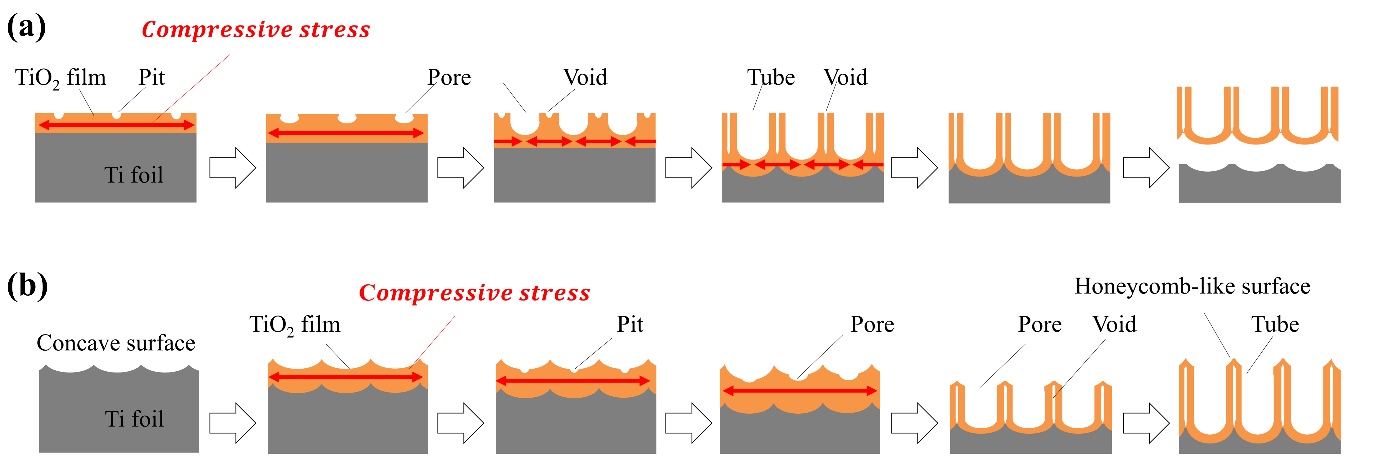


**Figure S5**. Schematic of formation of TiO_2_ nanotubes by (a) first and (b) second anodization steps.

The numbers and ratios of the defects are presented in Fig. S6 and S7. The majority of the coordination number was 6, and it was more than the sum of the counts of the other coordination numbers. In all samples, there were slightly more pores with the coordination number of 5 than those of the coordination number of 7. The limitation of the Voronoi diagram in a fixed image is that we cannot use pores at the boundary because there is no further point outside the image to be connected to points inside the image.


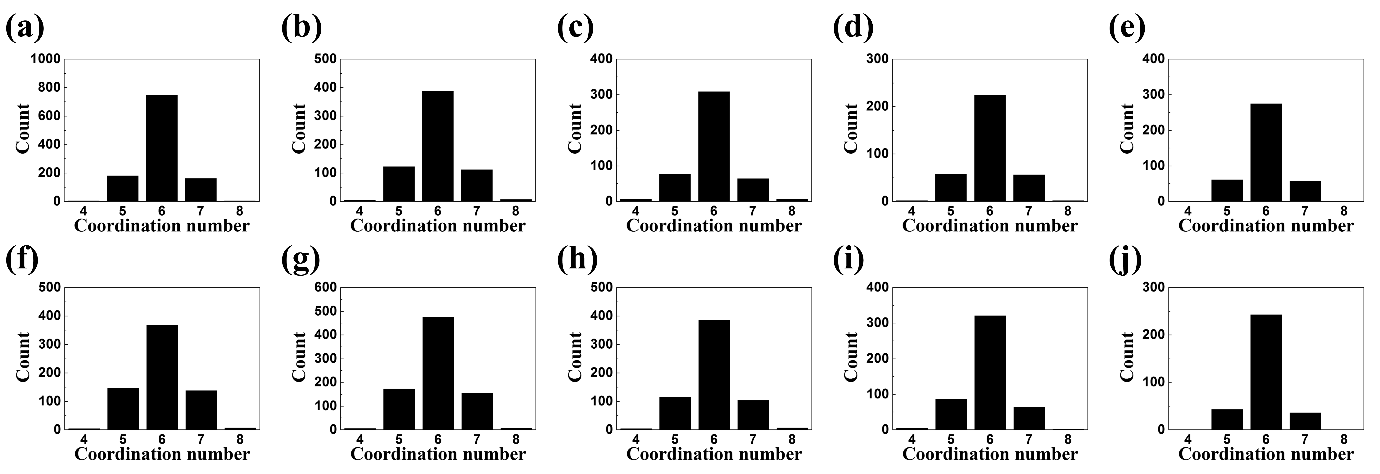


**Figure S6**. Counts of the coordination number of pores in TiO_2_ nanotube arrays anodized at 60 V for 1 hour in ethylene glycol containing (a – e) 2, 4, 6, 8, 10 vol % distilled water with 0.2 wt. % NH_4_F and (f – j) 2, 4, 6, 8, 10 vol. % distilled water with 0.4 wt. % NH_4_F.


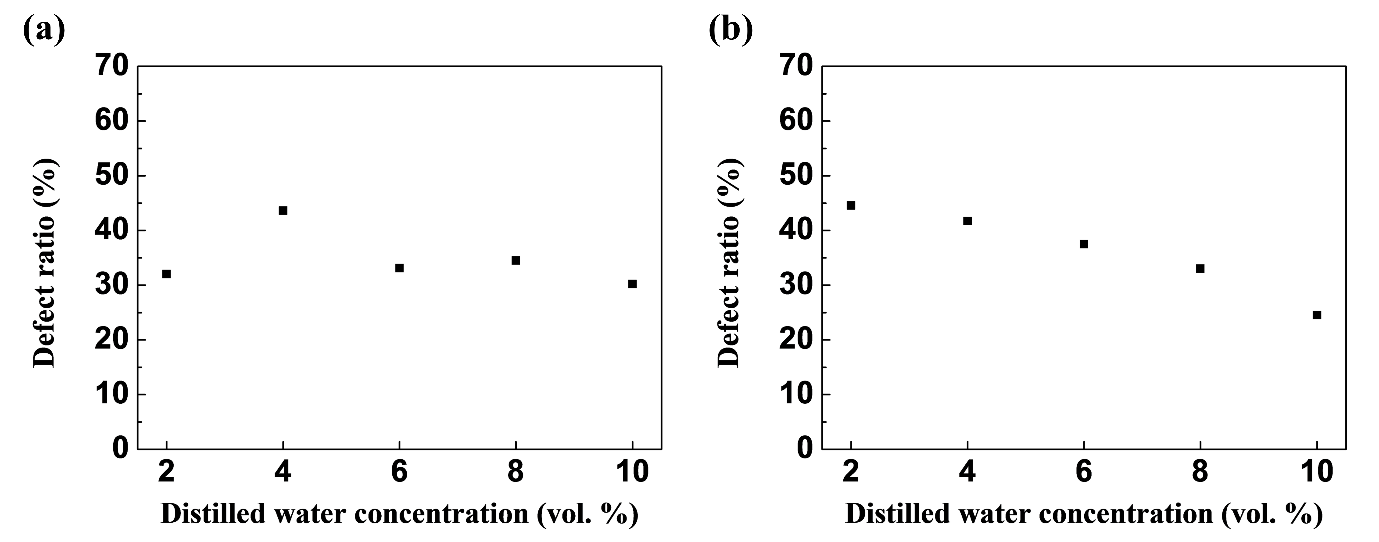


**Figure S7**. Defect ratios of TiO_2_ nanotube arrays anodized at 60 V for 1 hour in ethylene glycol containing (a) 0.2 wt. % NH_4_F and (b) 0.4 wt. % NH_4_F as a function of vol. % of distilled water.

We measured the length of the three diagonal lines of hexagonal rings in Fig. 3(a – j) as shown in Fig. S8(a – j) and calculated their relative standard deviation as shown in Fig. S9(a) and (b). From the relative standard deviation, we can determine which sample has the most uniform hexagonal FFT ring or which sample has the most distorted hexagonal FFT ring. The value of the relative standard deviation of all samples is smaller than 0.03. We found that the samples with 0.2 wt. % NH_4_F and 2 vol. % distilled water and with 0.4 wt. % NH_4_F and 8 vol. % distilled water have the smallest relative standard deviation, which means that they have the most undistorted hexagonal ring. Fig. S10(a – j) presents the normalized deviation of the three diagonal lines showing visually the comparison of the relative standard deviation. The most distorted hexagonal ring appears in the samples with 0.2 wt. % NH_4_F and 4 vol. % distilled water and with 0.4 wt. % NH_4_F and 2 vol. % distilled water.


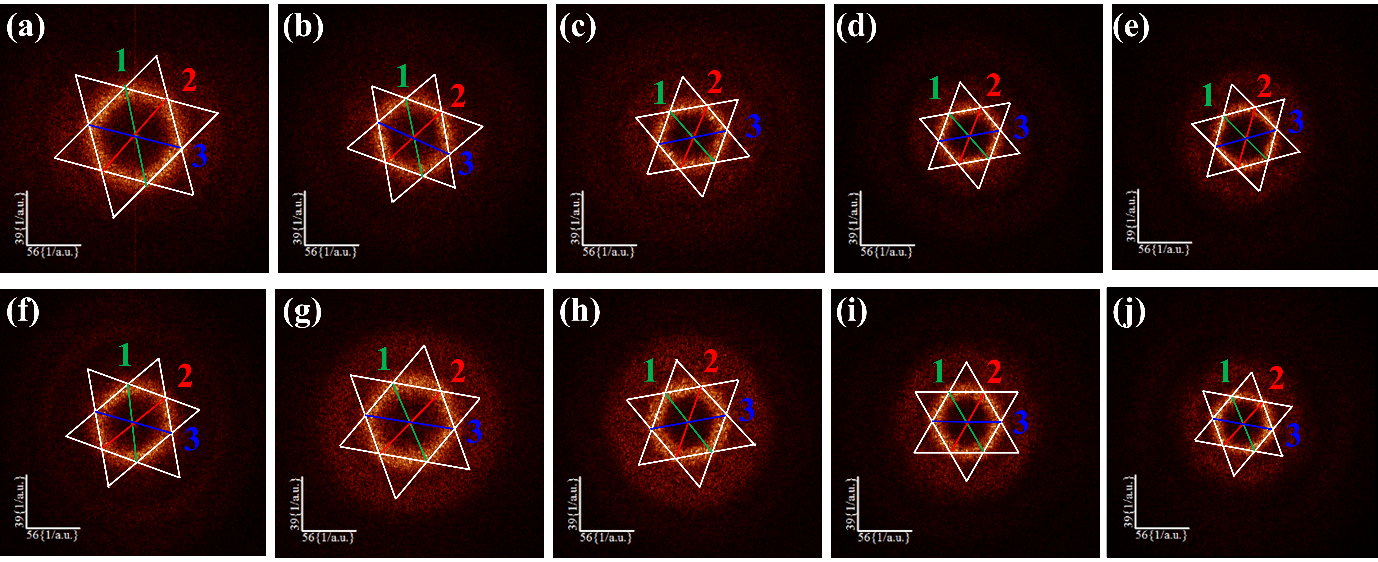


**Figure S8**. Three diagonal lines of fast Fourier transform radius profiles of TiO_2_ nanotube arrays anodized at 60 V for 1 hour in ethylene glycol containing (a – e) 2, 4, 6, 8, 10 vol. % distilled water with 0.2 wt. % NH_4_F and (f – j) 2, 4, 6, 8, 10 vol. % distilled water with 0.4 wt. % NH_4_F.


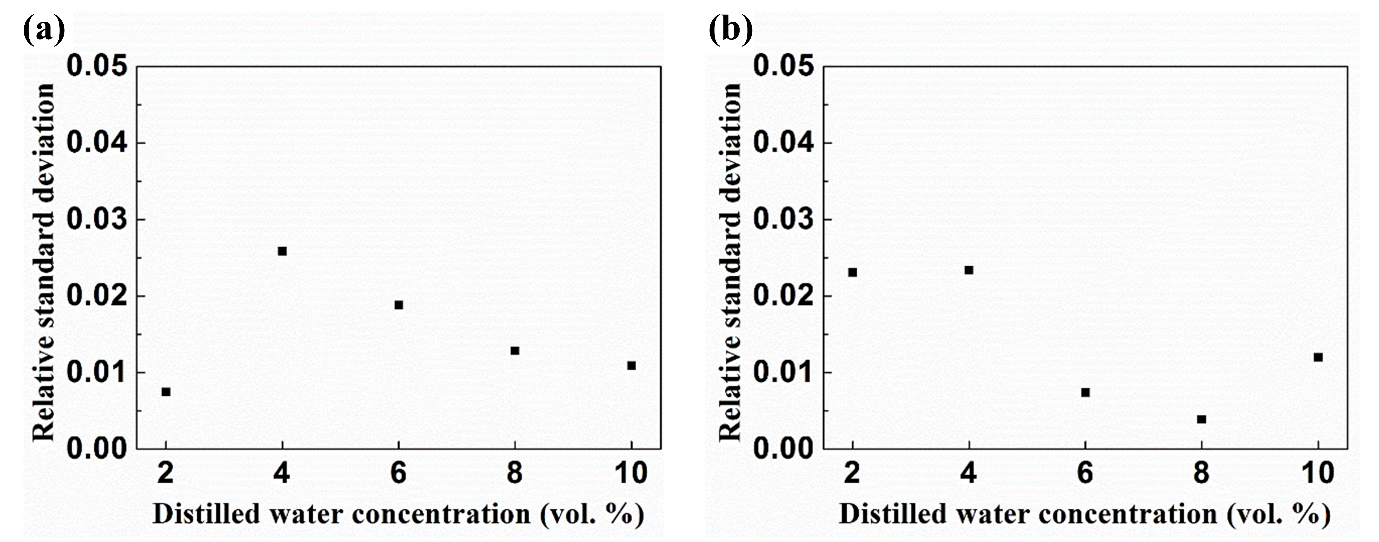


**Figure S9**. Relative standard deviation of three diagonal lines of fast Fourier transform radius profiles of TiO_2_ nanotube arrays at 60 V for 1 hour in ethylene glycol containing (a) 0.2 wt. % NH_4_F and (b) 0.4 wt. % NH_4_F as a function of vol. % of distilled water.


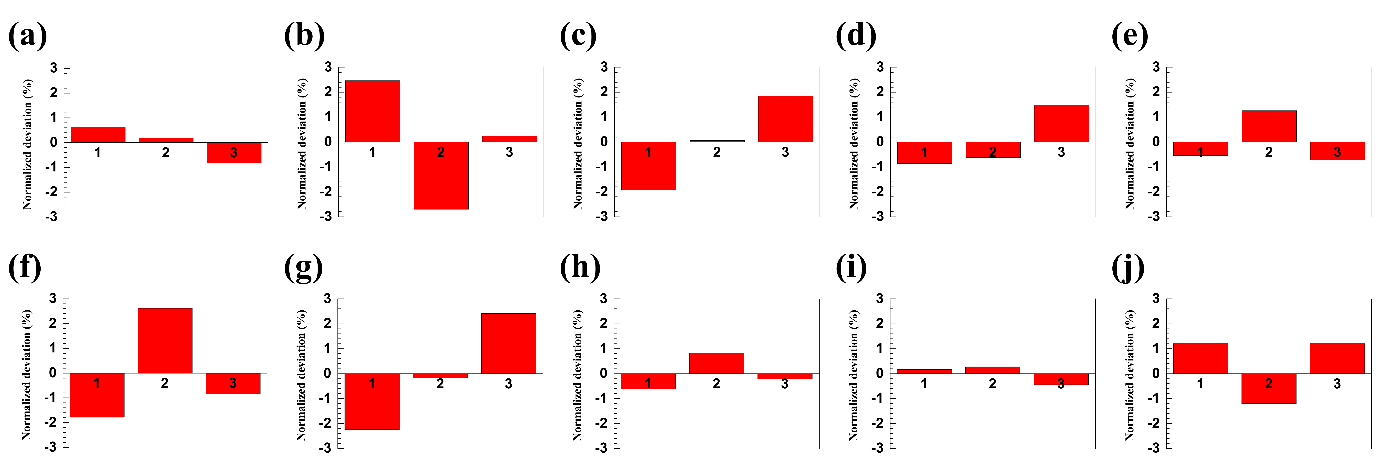


**Figure S10**. Normalized deviations of three diagonal lines of fast Fourier transform radius profiles of TiO_2_ nanotube arrays anodized at 60 V for 1 hour in ethylene glycol containing (a – b) 2, 4, 6, 8, 10 vol. % distilled water with 0.2 wt. % NH_4_F and (f – j) 2, 4, 6, 8, 10 vol. % distilled water with 0.4 wt. % NH_4_F.

Fig. S11(a – j) shows average fast Fourier transform (FFT) radius profiles of TiO_2_ nanotube arrays. We obtained the average FFT radius profiles using WSxM 5.0 Develop 8.2 software (Freeware). There is a peak in an average FFT radius profile, and a value of a ratio of the maximum intensity of the FFT radius profile ($H$) to its full width at half maximum ($W_{\frac{1}{2}}$, FWHM) is a criterion of regularity of an FFT image^9^.

We calculated regularity ratio (RR) from the average FFT radius profiles. The regularity ratio is an indicative of the degree to which the pores are evenly arranged. The regularity ratio was calculated by Equation (3) as reported in the prior report^9^.

RR = $\frac{H}{W_{\frac{1}{2}}\cdot D_{i}}$ (3)

Fig. S12(a) and (b) show the regularity ratio of the TiO_2_ nanotube arrays fabricated in 0.2 wt. % NH_4_F and 0.4 wt. % NH_4_F. The highest regularity ratio is presented in 0.2 wt. % NH_4_F and 10 vol. % distilled water and in 0.4 wt. % NH_4_F and 2 vol. % distilled water.


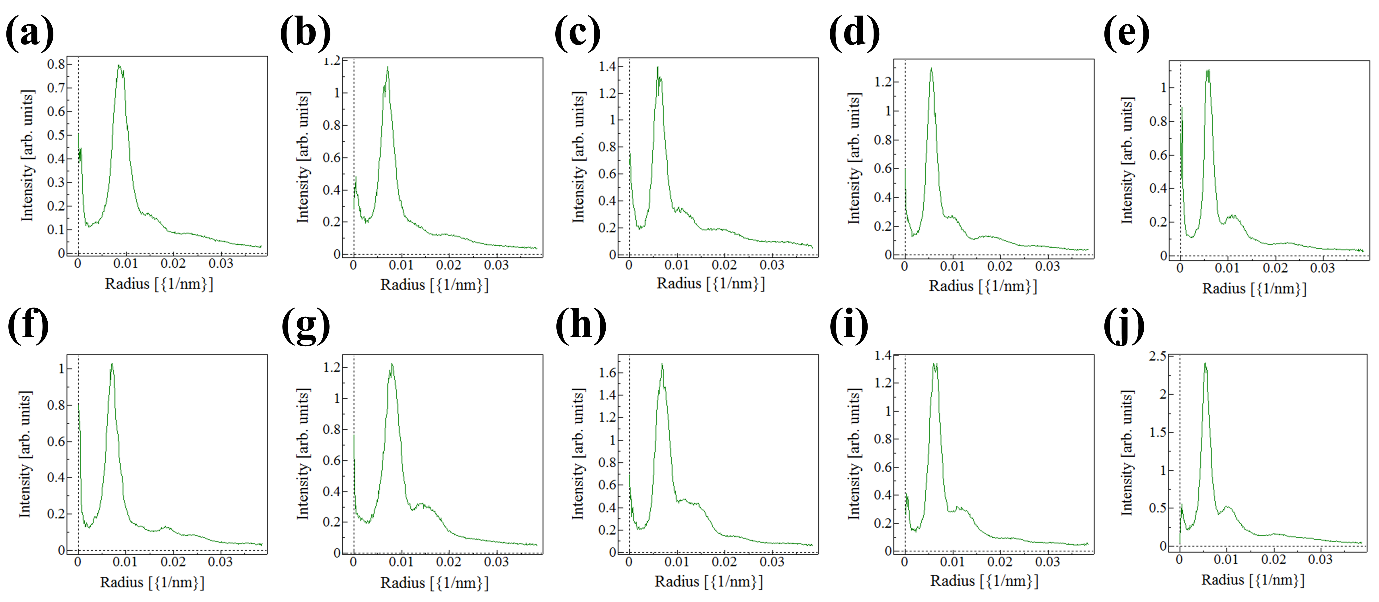


**Figure S11**. Average fast Fourier transform radius profiles of TiO_2_ nanotube arrays anodized at 60 V for 1 hour in ethylene glycol containing (a – e) 2, 4, 6, 8, 10 vol. % distilled water with 0.2 wt. % NH_4_F and (f – j) 2, 4, 6, 8, 10 vol. % distilled water with 0.4 wt. % NH_4_F.


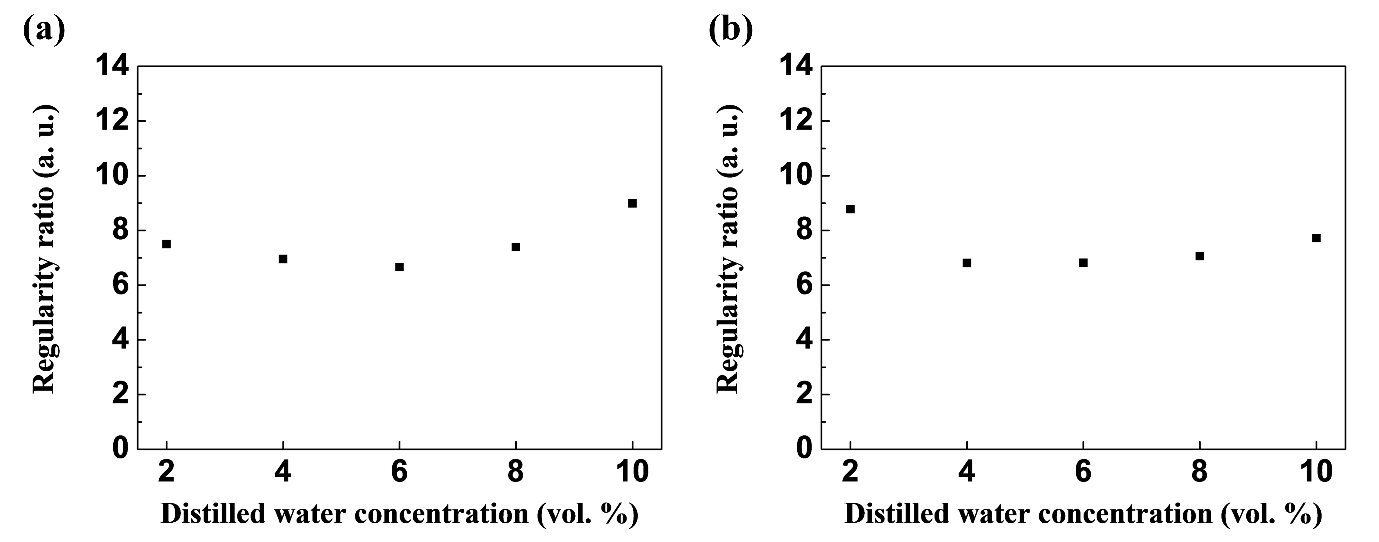


**Figure S12**. Regularity ratio of TiO_2_ nanotube arrays anodized at 60 V for 1 hour in ethylene glycol containing (a) 0.2 wt. % NH_4_F and (b) 0.4 wt. % NH_4_F as a function of vol. % of distilled water.

Fig. S13 represents the correlation between the ordering defects and the surface morphology of the TiO_2_ nanotube arrays. We derived defect ratios from regularity ratio and average diameter as a function of the concentration of water. H means higher defect ratio and L means lower defect ratio.


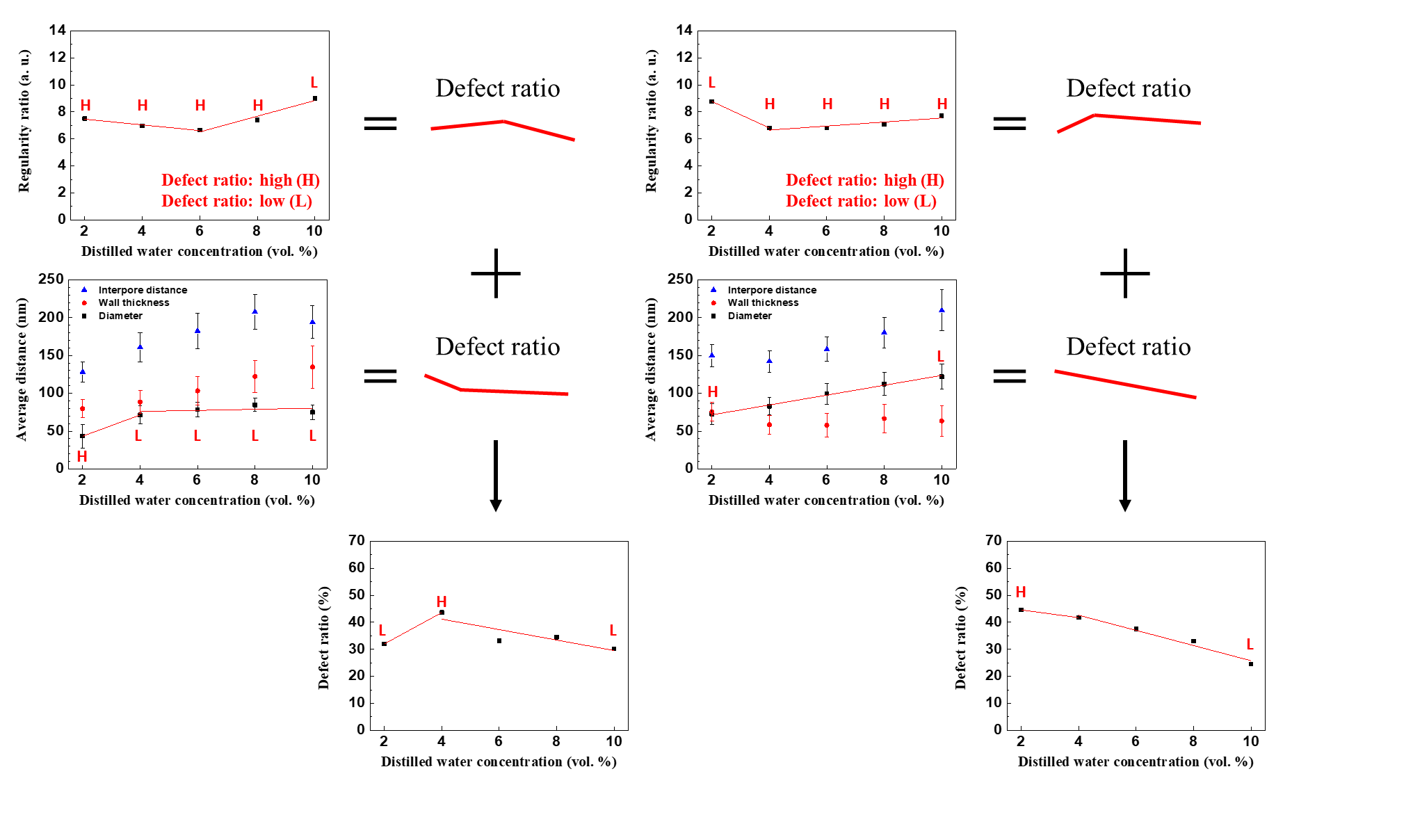


**Figure S13**. Schematic illustration of relationship between regularity ratio, pore diameter, and defect ratio.

We calculated the circularity (c) of the pores in the TiO_2_ nanotube arrays with Equation (4) using Image J 1.49v (Freeware) (see Fig. S14(a) and (b))^9,10^.

c = $4\pi\left( \frac{S}{{perimeter}^{2}} \right)$ (4)

where S is a surface area of a single pore.


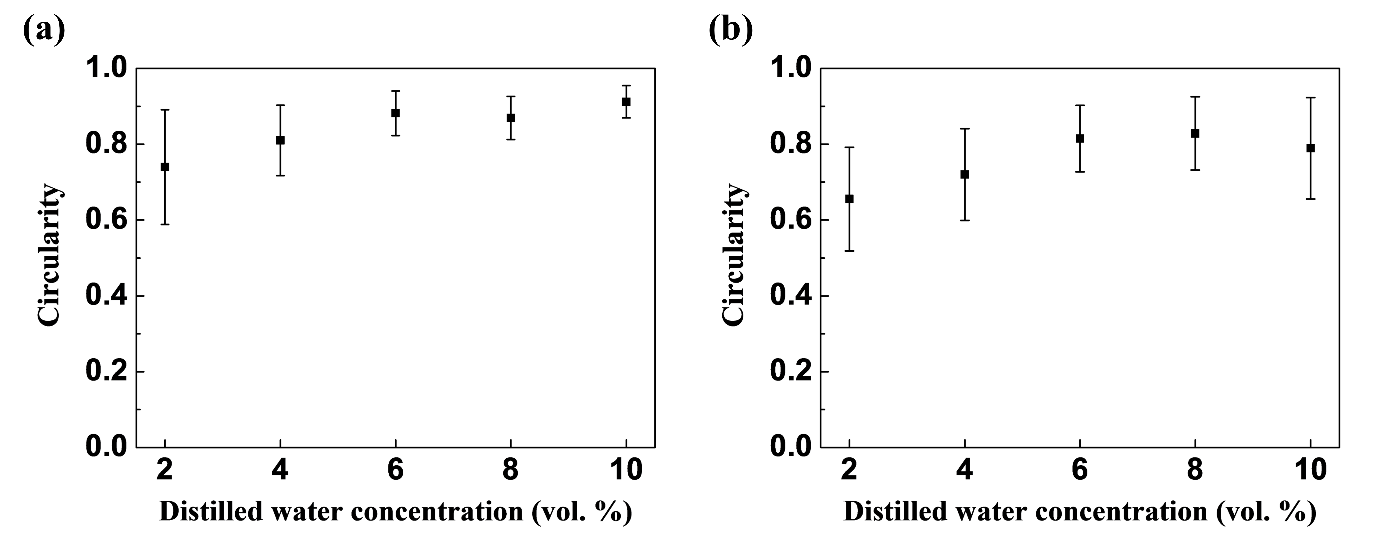


**Figure S14**. Circularity of pores in TiO_2_ nanotube arrays anodized at 60 V for 1 hour in ethylene glycol containing (a) 0.2 wt. % NH_4_F and (b) 0.4 wt. % NH_4_F as a function of vol. % of distilled water.

**References**

1. Raja, K. S., Gandhi, T. & Misra, M. Effect of water content of ethylene glycol as electrolyte for synthesis of ordered titania nanotubes. *Electrochem. Commun.* **9**, 1069-1076 (2007).

2. Sun, H. *et al.* Towards TiO_2_ nanotubes modified by WO_3_ species: influence of ex situ crystallization of precursor on the photocatalytic activities of WO_3_/TiO_2_ composites. *J. Phys. D: Appl. Phys.* **48**, 1-8 (2015).

3. Kojima, R., Kimura, Y., Bitoh, M., Abe, M. & Niwanoa, M. Investigation of Influence of Electrolyte Composition on Formation of Anodic Titanium Oxide Nanotube Films. *J. Electrochem. Soc.* **159**, D629-D636 (2012).

4. Ge, M. *et al.* One-dimensional TiO_2_ Nanotube Photocatalysts for Solar Water Splitting. *Adv. Sci.* **4**, 1600152 (2017).

5. Nishanthi, S. T., Iyyapushpam, S. & Padiyan, D. P. Role of Water Content in Anodization of Titanium to Fabricate TiO_2_ Nanotubes and its Properties, *International Conference on Advanced Nanomaterials & Emerging Engineering Technologies (ICANMEET-2013).*

6. Tsui, L.-k. & Zangari, G. Water content in the anodization electrolyte affects the electrochemical and electronic transport properties of TiO_2_ nanotubes: a study by electrochemical impedance spectroscopy. *Electrochim. Acta* **121**, 203-209 (2014).

7. Shin, Y. & Lee, S. Self-Organized Regular Arrays of Anodic TiO_2_ Nanotubes. *Nano Lett.* **8**, 3171-3173 (2008).

8. Meng, X. *et al.* Fabrication of Free Standing Anodic Titanium Oxide Membranes with Clean Surface Using Recycling Process. *J. Nanosci. Nanotechnol.* **10**, 4259-4265 (2010).

9. Zaraska, L., Stępniowski, W. J., Ciepiela, E. & Sulka, G. D. The effect of anodizing temperature on structural features and hexagonal arrangement of nanopores in alumina synthesized by two-step anodizing in oxalic acid. *Thin Solid Films* **534**, 155-161 (2013).

10. Zaraska, L., Stępniowski, W. J., Sulka, G. D., Ciepiela, E. & Jaskuła, M. Analysis of nanopore arrangement and structural features of anodic alumina layers formed by two-step anodizing in oxalic acid using the dedicated executable software. *Appl. Phys. A: Mater. Sci. Process.* **114**, 571-577 (2013).
